# Associations of Individual Characteristics and Socioeconomic Status With Heated Tobacco Product Harmfulness Perceptions in Japan: A Nationwide Cross-sectional Study (INFORM Study 2020)

**DOI:** 10.2188/jea.JE20230177

**Published:** 2024-09-05

**Authors:** Takumi Momosaka, Junko Saito, Aki Otsuki, Akiko Yaguchi-Saito, Maiko Fujimori, Aya Kuchiba, Kota Katanoda, Reo Takaku, Taichi Shimazu

**Affiliations:** 1 Division of Behavioral Sciences, National Cancer Center Institute for Cancer Control, Tokyo, Japan; 2 School of International and Public Policy, Hitotsubashi University, Tokyo, Japan; 3 Faculty of Human Sciences, Tokiwa University, Ibaraki, Japan; 4 Division of Survivorship Research, National Cancer Center Institute for Cancer Control, Tokyo, Japan; 5 Graduate School of Health Innovation, Kanagawa University of Human Services, Kanagawa, Japan; 6 Division of Biostatistical Research, Institution for Cancer Control/Biostatistics Division, Center for Research Administration and Support, National Cancer Center, Tokyo, Japan; 7 Division of Population Data Science, National Cancer Center Institute for Cancer Control, Tokyo, Japan

**Keywords:** heated tobacco product, INFORM Study, Japan

## Abstract

**Background:**

In Japan, heated tobacco products (HTPs) are promoted by the tobacco industry as reduced-risk tobacco products despite the lack of evidence for this claim. This study determined the distribution of HTP-harmfulness perception and identified the explanatory factors associated with the perception of HTP as less harmful than conventional cigarettes.

**Methods:**

A nationwide cross-sectional survey was conducted with Japanese people aged 20 years or older (INFORM Study 2020) using a self-administered questionnaire. We performed descriptive analysis and weighted logistic regression analysis to examine the relationship between explanatory factors (eg, individual characteristics, socioeconomic status, and trusted sources of cancer information) and the perception of HTPs as less harmful.

**Results:**

Among 3,420 participants, the proportions of those who perceived HTPs as less harmful were 40.3% and 18.3% for users and non-users of tobacco, respectively. For participants aged 20–39 years, the proportions were 49.9% and 30.4%, respectively. Among 1,160 tobacco non-users who were familiar with HTPs, male, aged under 39 years, and having lower education were associated with the perception of HTPs as less harmful. Trusted sources of cancer information were not associated with the perception of HTPs as less harmful.

**Conclusion:**

This study showed that, among tobacco non-users, being male, aged under 39 years, and having lower education were associated with a perception of HTPs as less harmful. Public health stakeholders should provide the latest evidence about HTP harmfulness in their daily practice and strengthen the regulations on HTP marketing directed at both tobacco- and tobacco non-users.

## INTRODUCTION

In recent years, new tobacco products, such as heated tobacco products (HTPs), have become prevalent worldwide. HTPs are electronic devices that heat tobacco leaves and generate nicotine-containing aerosol for inhalation.^1^ Since the launch of Phillip Morris International’s IQOS in Japan in 2014, the number of HTP users has been increasing. According to the 2019 National Health and Nutrition Survey in Japan, 26.7% of tobacco users in Japan used HTPs, and the rate was particularly high among young people.^2^

HTPs pose new tobacco control challenges; although the levels of some harmful and potentially harmful constituents of HTPs are lower than those of conventional cigarettes,^3^^,^^4^ there is little evidence on whether HTPs reduce disease risk.^5^ The United States Food and Drug Administration (FDA) has rejected “reduced risk” claims in marketing of HTPs marketing; however, it has permitted marketing with “reduced-exposure” claims.^6^ The tobacco industry continues to market HTPs as reduced-risk tobacco products,^7^^,^^8^ however, misleading consumers into believing that HTP use reduces disease risk compared to conventional cigarettes.^9^ After the FDA authorization, new advertisement themes have emerged, such as switching from conventional cigarettes and convenience (eg, indoor use).^10^ A survey of a nationally representative sample in Japan in 2018 showed that 47.5% of tobacco users perceived HTPs as less harmful than cigarettes.^11^

Tobacco non-users are also widely exposed to tobacco advertising in the media. The risk of second-hand aerosol smoke exposure may increase for tobacco non-users as HTP-user cohabitants may be allowed to smoke indoors. In Japan, 30.5% of tobacco non-users are exposed to HTP advertising in retail stores, 20% through television, newspapers, and posters, and 14% through the Internet.^12^ Thus, beliefs and perceptions of tobacco users and tobacco non-users about health risks have a huge impact on their behavior. These perceptions are not only key predictors of initiation and continuation of HTP use,^13^^,^^14^ they are also associated with HTP use in private or smoke-free public places.^15^

Previous studies have identified factors associated with perceived HTP harmfulness. Kim et al (2018) conducted an online survey to analyze explanatory factors among 7,000 Koreans individuals^16^ and found that HTP users aged 34 years or younger, male, and in the high-income category perceived HTPs as less harmful. The marketing of HTPs as “clean,” “high-tech,” “sophisticated,” and “safer” products compared to conventional cigarettes^17^^,^^18^ may attract young, male adults with a high socioeconomic status (SES),^19^ which may be related not only to individual characteristics but also to trust in media.^20^ Compared to tobacco non-users, tobacco users are more likely to trust information from media with variable accuracy, such as information published on the Internet, than information from health professionals.^21^

For these reasons, studies focusing on the perception of HTP harmfulness among populations that include tobacco non-users are necessary.^5^ However, to the best of our knowledge, limited studies have examined the factors related to HTP harmfulness perception, including trusted sources of cancer information, among both tobacco users and tobacco non-users.^11^^,^^16^ Moreover, since the diffusion and acceptance of HTPs varies from country to country,^18^ evidence from Japan would be vital for tobacco control in the country, where HTP use is the most prevalent but few laws prohibiting HTP advertising exist.^11^

Therefore, this study aims 1) to demonstrate the distribution of HTP harmfulness perception among tobacco users and tobacco non-users in Japan (Aim 1), and 2) to identify the explanatory factors (eg, individual characteristics, socioeconomic status, trusted sources of cancer information) associated with the perception of HTPs as less harmful using a nationwide health communication survey in Japan (Aim 2).

## METHODS

### INFORM Study 2020

We conducted the INFORM Study 2020, a nationally representative cross-sectional survey on health information access for consumers in Japan, to monitor cancer information seeking, knowledge, attitudes, beliefs, and behaviors. The self-administered questionnaire consisted of items from the Health Information National Trends Survey (HINTS) in the United States^22^ and also includes items related to health communication that are not covered in the HINTS but are relevant to Japan. The sampling for this survey is described in detail elsewhere.^23^ Briefly, 10,000 Japanese individuals aged 20 years or older were randomly sampled from 500 census areas using the Basic Resident Registration using a two-stage stratified random sampling method. The census areas were chosen from 35 strata in nine regions across Japan and four city sizes.

Data collection for the INFORM Study 2020 was done between August 1, 2020 and September 30, 2020. The invitation letter and questionnaire were sent via mail to 10,000 individuals and was undeliverable to 281 individuals. Of those 9,719 individuals, 3,605 consented to the survey and completed the questionnaire, resulting in a participation rate of 37.1%. In this study, we included 3,420 participants after excluding participants with missing data for the perception of HTP harmfulness, status of tobacco use, and type of tobacco products. To explore the relationships between explanatory factors and the perception of HTP harmfulness (Aim 2), 1,496 participants (1,160 tobacco non-users and 336 tobacco users) who had heard of/knew of HTPs and their harmfulness (ie, those who responded to HTP harmfulness compared to cigarettes as much less harmful/less harmful/as harmful/more harmful/much more harmful) were included.

### Perception of HTP harmfulness

The perception of HTP harmfulness was assessed by the following question: “What do you think of heated tobacco products (eg, PloomTech, IQOS, and glo) as compared to cigarettes?” The potential responses and clarifications were as follows: “They are much less harmful,” “They are less harmful,” (classified as less harmful); “They are as harmful,” “They are more harmful,” “They are much more harmful,” (classified as as/more harmful); “I don’t know about the harmfulness,” (classified as not knowing the harmfulness); “I have never heard of heated tobacco products,” and “I have heard of heated tobacco products before but don’t know them well” (classified as never heard of/don’t know HTPs well).

### Tobacco use behaviors

In this study, tobacco user is defined as a person reporting use of any type of tobacco product daily or less than daily at the time of survey. We classified tobacco product use into “tobacco users” (tobacco product use every day, use sometimes) and “tobacco non-users” (tobacco product used previously but have not used for than a month, never used). The “tobacco users” were also classified into “cigarette users” (those who smoked cigarettes or other types of tobacco but not HTPs) and “HTP users” (those who smoked HTPs or made dual/triple use of HTPs and other products), based on the type of tobacco products they used. The details are provided in eMaterial 1.

### Explanatory factors

The following potential explanatory factors for the perception of HTP harmfulness were used: socio-demographic factors (gender, age groups, and marital status), socio-economic factors (education, household income, and occupation), health communications (trusted sources of cancer information; ie, we asked how much they trusted different sources of information), tobacco use behaviors (types of tobacco use of the individuals and those living with them), and cancer history (cancer history of the individuals and their family). The details of categorization of each explanatory factor are shown in eMaterial 1.

### Statistical analyses

A weighted analysis was conducted to calculate accurate population parameter estimates to account for the complex sampling design and nonresponses for the Japanese general population. We calculated the weight for each participant by multiplying the sampling weight and the nonresponse weight. Following the sampling strategy of the survey, for each participant, the sampling weight was calculated as the reciprocal of the probability of selecting the participant for the survey in the stratum. It was assumed that the respondents in each nonresponse adjustment cell were a random sample of the samples in that cell^24^; accordingly, the nonresponse weight was estimated as the reciprocal of the proportion of respondents in “nonresponse adjustment cells.” A search algorithm^25^ was used to create the nonresponse adjustment cells based on the variables of the sampling strata, gender, and age group, which resulted in a total of 26 nonresponse adjustment cells. Specifically, based on our sampling strategy, individuals are selected from the population with a rough probability of 0.0001. Each selected subject can be considered to represent 10,000 (= 1/0.0001) individuals in the population, and they are assigned a sampling weight of 10,000 (actual sampling weights vary slightly for each of the 35 sampling strata, ranging from 10,291 to 10,457). We then considered nonresponse weights since not all selected individuals participated. The estimated nonresponse weights varied, ranging from a low of 1.86 to a high of 5.14, depending on the 26 nonresponse adjustment cells. For example, each female participant aged 40 years in a sampling stratum (region: Hokkaido, city size: towns and villages) is assigned a nonresponse weight 2.80. The final weights for those are a sampling weight of 10,313 times a nonresponse weight of 2.80.

We performed a descriptive analysis of perceived HTP harmfulness by types of tobacco use and age group among all the participants (*n* = 3,420), and each explanatory factor among participants who had heard of/knew about HTPs and their harmfulness (*n* = 1,496) for Aim 1. Then, we performed a logistic regression analysis among the same participants (*n* = 1,496) to calculate the odds ratio (OR) for Aim 2. The reason for limiting the part of the analysis to participants who had heard of/knew HTPs and their harmfulness was to exclude the influence of whether they were aware of HTP and to generate evidence that would contribute to interventions that would approach those who were aware of HTP and perceived HTP as not harmful. However, we also performed a sub-analysis that included participants who have not heard of/knew about HTPs and their harmfulness in the distribution of Aim 1.

The 95% confidence intervals (CIs) were estimated using the Taylor series linearization method.^26^ In the logistic regression analysis, the missing indicator method was applied for missing values.^27^ We stratified types of tobacco use for the analyses on main table. In addition, we showed the estimation results for “Crude” from a univariate analysis and “Adjusted” from a multivariate analysis using all the variables (ie, gender, age groups, marital status, education, household income, occupation, trusted sources of cancer information, types of tobacco use of the individuals and those living with them, and cancer history of the individuals and their family). Stata SE version 14.2 (StataCorp, College Station, TX, USA) was utilized with the svyset command, specified with 500 census areas as the variable of the primary sampling units and the 35 strata as the variable of strata on main table result.

As a sensitivity analysis, we conducted a logistic regression analysis by types of tobacco use (cigarette or HTP users) with 336 tobacco users without using the Taylor series linearization method due to the small sample size.

### Ethical approval

The INFORM Study 2020 protocol was approved by the Research Ethics Committee of the National Cancer Center (research project number: 2019-290).

## RESULTS

Among the 3,605 participants who consented to the survey, 3,420 participants (2,871 tobacco non-users and 549 tobacco users) were included in the descriptive analysis (Aim 1) after excluding participants with missing data for the perception of HTP harmfulness and status of tobacco use. Table 1 presents the distributions of perceived HTP harmfulness among the participants by types of tobacco use and age group. The percentages in the table were calculated by the percentage of the perceived HTP harmfulness among each age group. The proportion significantly differed in three age groups regarding the perception of HTPs among different tobacco product users (*P* < 0.0001).

**Table 1.** The distributions of the perceived HTP harmfulness by status of tobacco use and age group (*n* = 3,420)

	Tobacco non-users (*n* = 2,871)								Tobacco users (*n* = 549)							
	
	Less harmful		As/More harmful		Not knowing the harmfulness		Never heard/ Not knowing HTPs well		Less harmful		As/More harmful		Not knowing the harmfulness		Never heard/ Not knowing HTPs well	
	
	*n*	%	*n*	%	*n*	%	*n*	%	*n*	%	*n*	%	*n*	%	*n*	%
Age groups, years																
All age (*n* = 3,420)	495	(18.3)	665	(23.3)	507	(17.7)	1,204	(40.7)	216	(40.3)	120	(22.0)	123	(22.2)	90	(15.5)
Age 20–39 (*n* = 692)	174	(30.4)	171	(28.9)	102	(17.7)	138	(23.0)	53	(49.9)	23	(22.5)	25	(22.5)	6	(5.1)
Age 40–59 (*n* = 1,250)	179	(18.4)	272	(27.2)	201	(20.0)	357	(34.4)	106	(43.7)	58	(24.3)	48	(20.1)	29	(11.9)
Age 60 or older (*n* = 1,478)	142	(11.2)	222	(17.0)	204	(15.9)	709	(56.0)	57	(27.7)	39	(18.3)	50	(25.0)	55	(29.1)

HTP, heated tobacco product.

Participants with missing data for the perception of HTP harmfulness and status of tobacco use were excluded. The proportions shown in parentheses are weighted with the Taylor series linearization method. The percentages may not equal 100% due to rounding to the second decimal place. The percentages shown are the percentage of the perceived HTP harmfulness among each age group. “Tobacco non-users” were those who responded “tobacco product used previously but have not used for more than a month” or “never used” and “Tobacco users” were those who responded “tobacco product use every day” or “use sometimes.”

Table 2 shows the characteristics of 1,496 participants who had heard of/knew about HTPs and their harmfulness, stratified by types of tobacco use and perception of HTP harmfulness. The percentages in the table were calculated by the percentage of perceived HTP harmfulness among those who had each explanatory factor. For the tobacco non-users, around 50% of male, aged 20–39 years, and lower education (less than college) perceived HTPs as “Less harmful.” Conversely, among the tobacco users, 77.8% of the HTP users perceived HTPs as less harmful, while 55.1% of the cigarette users perceived HTPs as less harmful.

**Table 2.** The characteristics of participants who had heard of or knew HTPs and their harmfulness by status of tobacco use (*n* = 1,496)

	All (*n* = 1,496)				Tobacco non-users (*n* = 1,160)				Tobacco users (*n* = 336)			
		
	Less harmful		As/More harmful		Less harmful		As/More harmful		Less harmful		As/More harmful	
		
	*n*	%	*n*	%	*n*	%	*n*	%	*n*	%	*n*	%
Gender												
Male	382	(52.4)	363	(47.6)	217	(46.0)	272	(54.0)	165	(64.7)	91	(35.3)
Female	329	(44.3)	422	(55.7)	278	(42.1)	393	(57.9)	51	(64.7)	29	(35.3)
Age groups, years												
Age 20–39	227	(54.8)	194	(45.2)	174	(51.3)	171	(48.7)	53	(68.9)	23	(31.1)
Age 40–59	285	(47.1)	330	(52.9)	179	(40.4)	272	(59.7)	106	(64.3)	58	(35.7)
Age 60 or older	199	(43.6)	261	(56.4)	142	(39.6)	222	(60.4)	57	(60.3)	39	(39.8)
Marital status												
Married	498	(49.1)	539	(50.9)	338	(43.1)	458	(56.9)	160	(68.0)	81	(32.0)
No spouse	211	(47.9)	245	(52.1)	155	(45.5)	206	(54.5)	56	(57.1)	39	(42.9)
Missing	2	(59.0)	1	(41.0)	2	(59.0)	1	(41.0)	0	(0)	0	(0)
Education												
Less than college	319	(51.1)	304	(48.9)	211	(47.2)	237	(52.8)	108	(61.2)	67	(38.8)
College	171	(48.4)	198	(51.6)	133	(44.9)	178	(55.1)	38	(66.3)	20	(33.7)
University or higher	220	(46.3)	280	(53.7)	150	(40.1)	247	(60.0)	70	(69.3)	33	(30.7)
Missing	1	(21.4)	3	(78.6)	1	(21.4)	3	(78.6)	0	(0)	0	(0)
Household income												
Less than 4 million yen	200	(44.7)	253	(55.3)	137	(40.2)	210	(59.9)	63	(59.7)	43	(40.3)
4–8 million yen	305	(51.2)	303	(48.8)	216	(47.1)	252	(52.9)	89	(64.4)	51	(35.6)
More than 8 million yen	185	(48.6)	213	(51.4)	128	(43.1)	187	(56.9)	57	(68.1)	26	(32.0)
Missing	21	(59.7)	16	(40.3)	14	(49.8)	16	(50.2)	7	(100.0)	0	(0)
Occupations												
Non-workers	168	(44.2)	227	(55.8)	133	(41.4)	205	(58.6)	35	(61.9)	22	(38.1)
Manual	118	(53.1)	106	(46.9)	67	(49.4)	72	(50.6)	51	(59.2)	34	(40.8)
Lower-manual	225	(51.3)	221	(48.7)	156	(46.4)	184	(53.7)	69	(66.2)	37	(33.8)
Non-manual	191	(48.5)	217	(51.5)	133	(42.7)	191	(57.4)	58	(69.4)	26	(30.6)
Missing	9	(37.8)	14	(62.3)	6	(30.0)	13	(70.0)	3	(73.7)	1	(26.3)
Trusted sources of cancer information												
Healthcare professionals												
Trusting	489	(49.3)	533	(50.7)	344	(44.1)	460	(55.9)	145	(67.7)	73	(32.3)
Not trusting	203	(46.8)	242	(53.2)	136	(42.5)	197	(57.5)	67	(59.3)	45	(40.7)
Missing	19	(60.2)	10	(39.8)	15	(61.7)	8	(38.3)	4	(55.0)	2	(45.0)
Family or friends												
Trusting	277	(49.9)	285	(50.1)	195	(45.3)	244	(54.8)	82	(66.0)	41	(34.0)
Not trusting	417	(47.9)	488	(52.2)	287	(42.8)	413	(57.2)	130	(64.7)	75	(35.3)
Missing	17	(54.2)	12	(45.8)	13	(57.6)	8	(42.4)	4	(46.4)	4	(53.6)
Media												
Trusting	172	(47.5)	197	(52.5)	127	(42.8)	175	(57.2)	45	(67.4)	22	(32.6)
Not trusting	505	(48.8)	563	(51.2)	343	(44.0)	468	(56.0)	162	(63.9)	95	(36.1)
Missing	34	(54.9)	25	(45.1)	25	(51.8)	22	(48.3)	9	(67.7)	3	(32.3)
Internet												
Trusting	339	(50.0)	351	(50.0)	236	(44.8)	303	(55.2)	103	(67.9)	48	(32.1)
Not trusting	346	(47.3)	412	(52.7)	239	(42.5)	344	(57.5)	107	(62.6)	68	(37.4)
Missing	26	(55.0)	22	(45.1)	20	(55.0)	18	(45.0)	6	(54.7)	4	(45.3)
Government/Public Foundations												
Trusting	569	(48.5)	638	(51.6)	399	(43.3)	550	(56.7)	170	(66.8)	88	(33.2)
Not trusting	121	(49.2)	132	(50.8)	79	(45.3)	103	(54.7)	42	(58.7)	29	(41.3)
Missing	21	(55.0)	15	(45.0)	17	(56.5)	12	(43.5)	4	(48.5)	3	(51.5)
Types of tobacco use												
Tobacco non-users	495	(44.0)	665	(56.0)	495	(44.0)	665	(56.0)				
Cigarette users	112	(55.1)	90	(44.9)					112	(55.1)	90	(44.9)
HTP users	104	(77.8)	30	(22.2)					104	(77.8)	30	(22.2)
Types of tobacco use of people living with them												
Tobacco non-users	524	(47.4)	611	(52.6)	373	(42.9)	524	(57.2)	151	(64.1)	87	(35.9)
Cigarette users	99	(51.4)	100	(48.6)	58	(44.6)	79	(55.4)	41	(65.4)	21	(34.6)
HTP users	86	(55.4)	71	(44.6)	63	(51.8)	60	(48.2)	23	(69.1)	11	(30.9)
Missing	2	(48.1)	3	(52.0)	1	(45.3)	2	(54.7)	1	(52.1)	1	(47.9)
Cancer history (Their own)												
Yes	50	(48.8)	55	(51.2)	33	(41.0)	50	(59.0)	17	(78.3)	5	(21.8)
No	661	(48.7)	730	(51.3)	462	(44.2)	615	(55.8)	199	(63.9)	115	(36.1)
Cancer history (Their family)												
Yes	346	(45.7)	430	(54.3)	243	(40.6)	371	(59.4)	103	(64.7)	59	(35.3)
No	295	(50.1)	307	(49.9)	200	(45.1)	256	(54.9)	95	(65.0)	51	(35.0)
Not sure	15	(62.2)	10	(37.8)	10	(64.2)	7	(35.8)	5	(57.4)	3	(42.6)
Missing	55	(60.9)	38	(39.1)	42	(59.5)	31	(40.5)	13	(65.5)	7	(34.5)

HTP, heated tobacco product.

Participants who had not heard of/knew HTPs and their harmfulness, and who had missing data for the perception of HTP harmfulness were excluded. The proportions shown in parentheses are weighted with the Taylor series linearization method. The percentages may not equal 100% due to rounding to the second decimal place. The percentages shown were the percentage of the perceived HTP harmfulness among respondents who had each explanatory factor. “Tobacco non-users” were those who responded “tobacco product used previously but have not used for more than a month” or “never used” and “Tobacco users” were those who responded “tobacco product use every day” or “use sometimes.”

Table 3 shows the odds ratio of explanatory factors with the perception of HTP harmfulness through the weighted logistic regression analysis (Aim 2). Among tobacco non-users, those aged 40–59 and 60 years or older were less likely to perceive HTP as less harmfulness compared to the young people aged 20–39 years (OR 0.61; 95% CI, 0.44–0.83 and OR 0.56; 95% CI, 0.39–0.82, respectively), those who graduated from university or higher were less likely to perceive HTPs as less harmful compared to less than college (OR 0.65; 95% CI, 0.47–0.91), and those who were male were 1.33 times more likely to perceive HTP as less harmfulness compared to females (95% CI, 1.00–1.76). However, these relationships were not shown among tobacco users. Among tobacco users, HTP users were 3.05 times more likely than cigarette users to perceive HTPs as less harmful (95% CI, 1.75–5.32).

**Table 3.** Results of logistic regression by status of tobacco use: Association between explanatory factors and perceived HTP harmfulness (*n* = 1,496)

	Tobacco non-users (*n* = 1,160)				Tobacco users (*n* = 336)			
	
	Crude		Adjusted		Crude		Adjusted	
	
	Less harmful		Less harmful		Less harmful		Less harmful	
	OR	95% CIs	OR	95% CIs	OR	95% CIs	OR	95% CIs
Gender (reference: Female)								
Male	1.17	(0.92–1.50)	1.33	(1.00–1.76)	1.00	(0.57–1.75)	1.02	(0.50–2.07)
Age groups, years (reference: Age 20–39)								
Age 40–59	0.64	(0.48–0.86)	0.61	(0.44–0.83)	0.81	(0.46–1.42)	0.86	(0.47–1.58)
Age 60 or older	0.62	(0.46–0.84)	0.56	(0.39–0.82)	0.68	(0.37–1.28)	0.83	(0.37–1.84)
Marital status (reference: No spouse)								
Married	0.91	(0.70–1.18)	1.00	(0.74–1.34)	1.59	(0.98–2.59)	1.63	(0.95–2.79)
Education (reference: Less than college)								
College	0.91	(0.66–1.25)	0.87	(0.61–1.23)	1.24	(0.64–2.43)	1.07	(0.53–2.17)
University or higher	0.75	(0.56–1.00)	0.65	(0.47–0.91)	1.43	(0.83–2.47)	1.22	(0.59–2.52)
Household income (reference: Less than 4 million yen)								
4–8 million yen	1.33	(0.99–1.79)	1.26	(0.90–1.75)	1.22	(0.72–2.07)	0.93	(0.50–1.71)
More than 8 million yen	1.13	(0.81–1.57)	1.20	(0.83–1.74)	1.44	(0.78–2.66)	0.93	(0.43–2.01)
Occupations (reference: Non-workers)								
Manual	1.38	(0.92–2.09)	1.27	(0.82–1.97)	0.89	(0.44–1.82)	0.71	(0.33–1.55)
Lower-manual	1.22	(0.90–1.67)	1.30	(0.93–1.83)	1.21	(0.61–2.38)	1.01	(0.47–2.17)
Non-manual	1.05	(0.76–1.46)	1.15	(0.78–1.70)	1.40	(0.68–2.90)	1.07	(0.45–2.55)
Trusted sources of cancer information								
Healthcare professionals	1.07	(0.84–1.36)	1.00	(0.75–1.33)	1.44	(0.92–2.27)	1.21	(0.69–2.10)
Family or friends	1.10	(0.86–1.41)	1.10	(0.83–1.45)	1.06	(0.65–1.73)	0.91	(0.51–1.63)
Media	0.95	(0.72–1.26)	0.87	(0.62–1.23)	1.17	(0.66–2.08)	0.87	(0.42–1.83)
Internet	1.10	(0.86–1.39)	1.23	(0.91–1.65)	1.26	(0.80–1.98)	1.39	(0.81–2.39)
Government/Public Foundations	0.92	(0.66–1.30)	0.95	(0.64–1.41)	1.42	(0.80–2.51)	1.34	(0.67–2.66)
Types of tobacco use (reference: Cigarette users)								
HTP users					2.85	(1.75–4.67)	3.05	(1.75–5.32)
Types of tobacco use of people living with him/her (reference: Tobacco non-users)								
Cigarette users	1.07	(0.75–1.55)	0.95	(0.64–1.42)	1.06	(0.59–1.92)	1.40	(0.69–2.87)
HTP users	1.44	(0.99–2.09)	1.35	(0.89–2.04)	1.26	(0.55–2.85)	0.85	(0.32–2.28)
Cancer history (Their own) (reference: No)								
Yes	0.88	(0.55–1.39)	1.12	(0.68–1.86)	2.03	(0.77–5.37)	2.64	(0.88–7.91)
Cancer history (Their family) (reference: No)								
Yes	0.83	(0.65–1.06)	0.88	(0.68–1.13)	0.99	(0.61–1.60)	1.08	(0.63–1.87)
Not sure	2.18	(0.74–6.43)	2.10	(0.70–6.27)	0.73	(0.16–3.40)	0.92	(0.16–5.48)

CI, confidence interval; HTP, heated tobacco product; OR, odds ratio.

Participants who had not heard of/knew HTPs and their harmfulness, and who had missing data for the perception of HTP harmfulness and status of tobacco use were excluded. Missing values are controlled in all the models when existing. Standard errors are obtained from the Taylor linearized variance estimation. “Tobacco non-users” were those who responded “tobacco product used previously but have not used for more than a month” or “never used” and “Tobacco users” were those who responded “tobacco product use every day” or “use sometimes.”

eTable 1 shows the OR of explanatory factors with the perception of HTP harmfulness among tobacco users by types of tobacco products in the sensitivity analysis, and eTable 2 and eTable 3 show the characteristics of all the participants including those who have not heard of/knew about HTPs and their harmfulness (see eMaterial 2 for details of the explanatory factors).

## DISCUSSION

This study analyzed 3,605 Japanese individuals aged 20 years or older, in Japan, where HTP use is prevalent. A total of 18.3% and 40.3% of tobacco non-users and tobacco users, respectively, perceived HTPs as less harmful than conventional. Tobacco non-users and users aged 20–39 years accounted for 30.4% and 49.9% of this group, respectively. Among 1,160 tobacco non-users who had heard of/knew about HTPs and their harmfulness, those who were male, under 39 years old, and had lower education levels tended to perceive HTPs as less harmful. These relationships were not found for tobacco users.

Among tobacco smokers, previous studies in Japan reported that individual characteristics and SES, such as being male, younger than 34 years, having a higher income, frequent HTP use, and exposure to HTP advertisements on media, were associated with perceiving HTP as less harmful^11^; however, these studies did not consider tobacco non-users in their analyses. Among tobacco users, a study in Korea showed that HTP users, those aged less than 34 years, males, and having higher income levels were associated with a perception of HTPs as less harmful, but associated factors for tobacco non-users are unknown.^16^

This study revealed that those who were male, aged under 39 years, or had lower education level (less than college) were associated with a perception of HTPs as less harmful among only tobacco non-users. This difference may occur because of both groups’ optimism regarding their tobacco use. Tobacco users tend to underestimate their relative risk of developing cancer compared to both other users and to tobacco non-users.^28^ This tendency may be a common characteristics of tobacco users, so there may not be a significant relationship between explanatory factors and a perception of HTPs as less harmful among tobacco users. Another possible reason is the differences in the impact of marketing. The recent HTPs adopt non-traditional marketing strategies, such as the “bait and hook” pricing strategy, social media marketing techniques, and sales through multiple channels, including dedicated HTP stores, retail outlets, and e-commerce sites.^29^ In addition, the tobacco industry markets HTPs to young males as clean and sophisticated alternatives to conventional cigarettes with less odor and harmfulness.^8^^,^^18^ Although tobacco non-users are generally well aware of the risks of tobacco use even when exposed to tobacco marketing,^30^ the marketing messages may have a greater impact, particularly on populations vulnerable to tobacco marketing, such as young males or those with lower education.

HTP users were more likely than conventional tobacco users to perceive HTPs as less harmful. This result was consistent with previous studies.^11^^,^^14^^,^^15^^,^^31^^,^^32^ The major reason for using HTPs in Japan was that they are less harmful for self and others.^33^ Several studies have reported lower levels of toxic substances in HTPs^4^^,^^34^^,^^35^; however, being less harmful does not justify their acceptance, as there is little evidence on whether HTPs reduce disease risks.^5^ However, the marketing claims that HTPs are less harmful than conventional tobacco products mislead consumers into believing that HTPs pose a lower health risk.^36^^,^^37^ Therefore, dissemination of the current latest evidence about HTPs and strengthening the regulations on HTP marketing in response to non-traditional marketing directed at both tobacco non-users and tobacco users are strongly needed, which may prevent the re-normalization of HTP use.

This study also found that there were no associations of income or occupations with the perceived HTP harmfulness for both tobacco non-users and tobacco users. Although those with high SES tend to use HTPs,^19^^,^^38^ it may be that higher prices prevent lower-income tobacco users from migrating from cigarettes to HTPs. However, their perceptions of HTPs as less harmful does not differ with high SES groups.^39^^,^^40^ Further, contrary to our hypothesis, particularly the significant association of trust in healthcare professionals with a perception of HTPs as more harmful, this study did not reveal any association between specific trusted information sources and harmfulness perception. In terms of healthcare professionals, they may not have provided sufficient information about the health risk involved in HTP use. Regarding media exposure, while the evidence shows that media contributes to lower harmfulness perception,^11^ other factors, like interesting content or attractive messages, which were not considered in this study, may cause HTPs to be perceived as less harmful.

Our results suggest that advocacy for tobacco control by public health stakeholders, such as anti-smoking campaigns, should target tobacco non-users as well as tobacco users. Providing scientific information to tobacco non-users regarding risks is crucial because a perception of HTPs as less harmful is associated with lower support for various HTP control regulations, including bans on promotion, advertisements, and use in smoke-free areas.^41^ Healthcare professionals, such as physicians, nurses, and pharmacists, would be key persons to provide the latest evidence to tobacco users and their non-tobacco user family members regarding HTP harmfulness, as simple advice by physicians and multiple health professionals has an effect to make users quit.^42^^,^^43^

This study’s strength is its weighted logistic regression analysis using nationally representative data from Japan, where HTP use is prevalent. To the best of our knowledge, there are limited studies on the HTP harmfulness perception of the Japanese population. Through our analysis, we determined the influence of demographic factors, such as age, gender, educational attainment, and smoking status, on HTP harmfulness perception. However, this study also has a few limitations. First, since a cross-sectional analysis does not identify causal effect, the present study did not reveal a causal relationship between the perception of HTP harmfulness and related explanatory factors, such as trusted information sources, types of tobacco use, and types of tobacco use of people living with them. Thus, further research is necessary to identify the appropriate causal effect in the future. Second, regarding a perception of HTPs as “less harmful,” it was not possible to distinguish between the perception that HTPs contain fewer harmful substances than cigarettes and the perception that HTPs are much less harmful in terms of disease risk, so there is no reason to quit. Therefore, the perception of HTPs as “less harmful” as a wrong perception would be misleading. Third, the present study did not determine the impact of HTP marketing exposure for the harmfulness perception in the analysis due to the lack of the items in the questionnaire. Fourth, the question about trusted information sources was limited to “information about cancer.” This variable was used as a proxy measure for responses about trust in general health information (including tobacco use). Although the overwhelming majority of people including tobacco users have a belief that tobacco use causes cancer,^44^^,^^45^ limiting the information to cancer may have resulted in a different distribution of responses. Future analyses with responses about sources of trusting information about tobacco use need to be conducted to see if the results differ. Fifth, although cognitive debriefing was conducted for people who were not researchers or healthcare professionals in the development of the questionnaire,^23^ self-administrated questionnaire may cause respondents to misunderstand the questions and write inaccurate answers.^46^ Sixth, the study’s sample size was limited in the sensitivity analysis. Since the number of HTP users living with other HTP users is quite small (*n* = 134), the results may have a lower statistical power, and interpretation must be done cautiously. Finally, as data were collected during the coronavirus disease 2019 (COVID-19) pandemic, the responses for types of tobacco use and perception of HTP harmfulness among participants might have been influenced by the pandemic, such as people with high-risk factors for COVID-19, would have probably quit smoking.^47^

### Conclusions

This study found that being male, aged under 39 years, and having less than college education were associated with the perception that HTPs were less harmful only among tobacco non-users. Moreover, the use of HTPs in tobacco users was associated with harmfulness perception. However, no association was found between trusted sources of cancer information and the HTP harmfulness perception for either tobacco non-users and tobacco users. It is important for public health stakeholders to provide the latest scientific evidence about HTP harmfulness in their daily practice; that is, although there are lower levels of some harmful and potentially harmful constituents in HTPs than in conventional cigarettes, there is a lack of evidence on the effects of HTPs’ disease risk reduction, and regulations on HTP marketing directed at both tobacco non-users and tobacco users should be strengthened.

## SUPPLEMENTARY MATERIAL

eMaterial 1. The details of categorization of each explanatory factor

eTable 1. Results of logistic regression by types of tobacco use: Association between explanatory factors and perceived HTP harmfulness (n = 336)

eTable 2. The characteristics of all the participants (n = 3,420)

eTable 3. The characteristics of all the participants by status of tobacco use (n = 3,420)
