# Supplementary material for: Associations of Individual Characteristics and Socioeconomic Status With Heated Tobacco Product Harmfulness Perceptions in Japan: A Nationwide Cross-sectional Study (INFORM Study 2020)
Source: J Epidemiol. 2024 Sep 5;34(9):411–8. doi: 10.2188/jea.JE20230177 (PMC11330704; doi:10.2188/jea.JE20230177)
Supplement: Supplementary file 1 [file je-34-411-s001.pdf]

eMaterial 1. The details of categorization of each explanatory factor

Socio-demographic factors

Marital status was divided into two categories: “Married” or “No spouse” (never married, widowed, divorced). Age was divided into three categories: 20–39 years, 40–59 years, and 60 years or older. Considering that heated tobacco products (HTPs) are prevalent among youth,[1] we defined individuals aged 20–39 years as young people, 40–59 years as middle-aged people, and 60 years or older as older people.

Socio-economic factors

Education was classified into three categories: “Less than college,” “College” (vocational school, college, technical college), and “University or higher.” Occupations were classified into four categories: “Non-workers” (none, housewife/husband, student), “Manual” (security, agriculture, forestry, fishery, production, processing, transportation, machine operation, construction, mining, shipment, cleaning, and packing jobs, among others), “Lower-manual” (eg, office clerk, sales, service), and “Non-manual” (eg, specialist, technician) based on the Japan Standard Occupational Classification, which is profiled by the Ministry of Internal Affairs and Communications that classifies

occupations into different groups.[2]

Health communications

Degree of trusted sources of cancer information was assessed as a proxy measure for responses about trust in general health information including tobacco product use, for the following nine sources with four responses ("Not at all," "A little," "Some," and "A lot"): "Physicians," "Healthcare professionals other than physicians," "Family or friends," "Newspapers or magazines," "Radio," "Internet," "Television," "Government health agencies (eg, Ministry of Health, Labour and Welfare, National Cancer Center)," "Public Interest Incorporated Foundations (eg, Japan Cancer Society, Foundation for Promotion of Cancer Research)."

The responses were categorized into "Not trusting" (for "Not at all" and "A little") and "Trusting" ("Some" and "A lot") for the nine information sources. The sources were classified as either "Healthcare professionals" (for "Physicians" and "Healthcare professionals other than physicians"), "Media" (for "Newspapers or magazines," "Radio," and "Television"), or "Government/Public Foundations" (for "Government health agencies" and "Public Interest Incorporated Foundations").

Tobacco use behaviors

Tobacco product use was classified into two categories: “Tobacco users” (tobacco product use every day, use sometimes) and “Non-tobacco users” (tobacco product used previously but have not used for than a month, never used). The “Tobacco users” were further classified into two categories according to the type of tobacco products used: “Cigarette users” and “HTP users”. Those who smoked cigarettes or other types of tobacco but not HTPs were defined as “Cigarette users”, while those who smoked HTPs or made dual/triple use of HTPs and other products were defined as “HTP users”. Similarly, the participants were asked about the types of tobacco use of persons cohabiting with them as well as about the type of tobacco their cohabitants used.

Cancer history

The cancer history of the participants was classified into two categories: “Yes” and “No,” based on whether they had a cancer diagnosis before and that of their family was classified into three categories: “Yes,” “No,” and “Not sure.”

REFERENCES

1. Tabuchi T, Shinozaki T, Kunugita N, Nakamura M, Tsuji I. Study Profile: The Japan "Society and New Tobacco" Internet Survey (JASTIS): A Longitudinal Internet Cohort Study of Heat-Not-Burn Tobacco Products, Electronic Cigarettes, and Conventional Tobacco Products in Japan. *J Epidemiol*. 2019;29(11):444–450. doi:10.2188/jea.JE20180116.
2. Tomioka K, Kurumatani N, Saeki K. The Association Between Education and Smoking Prevalence, Independent of Occupation: A Nationally Representative Survey in Japan. *J Epidemiol*. 2020;30(3):136–142. doi:10.2188/jea.JE20180195.

eTable 1. Results of logistic regression by types of tobacco use: Association between explanatory factors and perceived HTP harmfulness (n=336)

	Cigarette users (n=202)				HTP users (n=134)			
	Crude		Adjusted		Crude		Adjusted	
	Less harmful		Less harmful		Less harmful		Less harmful	
	OR	95% CI	OR	95% CI	OR	95% CI	OR	95% CI
Gender (Ref. Female)								
Male	1.03	(0.54 – 1.95)	1.16	(0.51–2.67)	0.88	(0.32–2.41)	1.06	(0.22–5.03)
Age groups (Ref. Age 20–39 years)								
Age 40–59 years	1.26	(0.56–2.83)	1.22	(0.47–3.19)	0.65	(0.25–1.66)	0.66	(0.20–2.22)
Age 60 years or older	1.13	(0.49–2.61)	1.25	(0.45–3.48)	0.69	(0.20–2.44)	0.33	(0.06–1.72)
Marital status (Ref. No spouse)								
Married	1.66	(0.91–3.03)	1.57	(0.76–3.27)	0.71	(0.26–1.93)	1.36	(0.37–5.01)
Education (Ref. Less than college)								
College	0.83	(0.37–1.85)	0.90	(0.36–2.24)	1.46	(0.47–4.57)	2.16	(0.47–9.92)
University or higher	1.24	(0.65–2.38)	1.21	(0.53–2.77)	1.07	(0.43–2.64)	0.73	(0.20–2.63)
Household income (Ref. Less than 4 million yen)								
4–8 million yen	1.28	(0.68–2.42)	1.05	(0.49–2.23)	0.65	(0.23–1.88)	0.61	(0.17–2.13)
More than 8 million yen	1.63	(0.76–3.49)	1.18	(0.46–3.02)	0.74	(0.23–2.35)	0.70	(0.16–3.07)
Occupations (Ref. Non-workers)								
Manual	1.21	(0.52–2.78)	1.36	(0.50–3.72)	0.41	(0.10–1.74)	0.10	(0.01–1.03)
Lower-manual	1.17	(0.52–2.63)	1.40	(0.51–3.82)	0.82	(0.19–3.51)	0.29	(0.03–2.63)
Non-manual	1.71	(0.71–4.09)	1.92	(0.61–6.02)	0.60	(0.14–2.56)	0.18	(0.02–1.78)
Trusted sources of cancer information								
Healthcare professionals	1.59	(0.89–2.85)	1.24	(0.61–2.53)	0.80	(0.32–1.99)	1.17	(0.33–4.16)
Family or friends	1.88	(1.03–3.42)	1.68	(0.83–3.42)	0.39	(0.17–0.92)	0.31	(0.11–0.86)
Media	1.56	(0.77–3.18)	0.88	(0.34–2.27)	0.74	(0.27–1.98)	0.44	(0.10–1.88)
Internet	1.63	(0.92–2.88)	1.44	(0.72–2.88)	1.24	(0.53–2.89)	2.11	(0.64–6.95)
Government/Public Foundations	1.94	(0.95–3.97)	1.45	(0.63–3.35)	0.94	(0.36–2.46)	0.82	(0.21–3.27)
Types of tobacco use of people living with him/her (Ref. Non-tobacco users)								
Cigarette users	1.29	(0.66–2.53)	1.29	(0.60–2.80)	1.86	(0.39–8.86)	1.69	(0.24–11.81)
HTP users	1.58	(0.50–4.96)	1.18	(0.29–4.82)	0.67	(0.23–1.94)	0.19	(0.04–1.01)
Cancer history (Their own) (Ref. No)								
Yes	1.88	(0.56–6.31)	1.92	(0.46–8.01)	2.42	(0.29–20.13)	3.24	(0.31–33.65)
Cancer history (Their family) (Ref. No)								
Yes	0.85	(0.48–1.53)	0.88	(0.44–1.78)	1.14	(0.49–2.64)	1.14	(0.41–3.16)
Not sure	1.09	(0.17–6.84)	2.09	(0.25–17.60)	0.64	(0.05–7.56)	0.52	(0.03–9.42)

CI, confidence interval; HTP, Heated tobacco product; OR, Odds ratio.
Participants who had not heard of/knew HTPs and their harmfulness, and who had missing data for the perception of HTP harmfulness and status of tobacco use were excluded. Missing values are controlled in all the models when existing. Standard errors are obtained from the Taylor linearized variance estimation. “Non-tobacco users” were those who responded “tobacco product used previously but have not used for than a month” or “never used” and “Tobacco users” were those who responded “tobacco product use every day” or “use sometimes.” “Tobacco users” were also classified into “Cigarette users” (those who smoked cigarettes or other types of tobacco but not HTPs) and “HTP users” (those who smoked HTPs or made dual/triple use of HTPs and other products) according to the type of tobacco product they use.

eTable 2. The characteristics of all the participants (n=3,420)

	All (n=3,420)							
	Less harmful		As/More harmful		Not knowing the harmfulness		Never heard/ Not knowing HTPs well	
	n	%	n	%	n	%	n	%
Gender								
Male	382	(26.1)	363	(23.7)	315	(20.0)	504	(30.3)
Female	329	(17.9)	422	(22.5)	315	(17.0)	790	(42.7)
Age groups								
Age 20–39 years	227	(33.7)	194	(27.8)	127	(18.5)	144	(20.0)
Age 40–59 years	285	(23.7)	330	(26.6)	249	(20.1)	386	(29.7)
Age 60 years or older	199	(13.3)	261	(17.2)	254	(17.0)	764	(52.6)
Marital status								
Married	498	(22.0)	539	(22.8)	438	(18.2)	921	(37.0)
No spouse	211	(21.9)	245	(23.8)	191	(19.0)	367	(35.3)
Missing	2	(19.4)	1	(13.5)	1	(11.8)	6	(55.2)
Education								
Less than college	319	(20.3)	304	(19.4)	295	(18.8)	685	(41.6)
College	171	(23.3)	198	(24.8)	131	(16.3)	286	(35.6)
University or higher	220	(23.7)	280	(27.4)	203	(19.6)	319	(29.4)
Missing	1	(10.2)	3	(37.3)	1	(12.0)	4	(40.6)
Household income								
Less than 4 million yen	200	(17.0)	253	(21.0)	214	(17.4)	566	(44.6)
4–8 million yen	305	(24.1)	303	(23.0)	257	(19.5)	458	(33.3)
More than 8 million yen	185	(25.8)	213	(27.3)	146	(19.2)	224	(27.7)
Missing	21	(23.9)	16	(16.1)	13	(12.4)	46	(47.6)
Occupations								
Non-workers	168	(14.4)	227	(18.1)	205	(16.8)	633	(50.8)
Manual	118	(25.0)	106	(22.1)	94	(20.4)	174	(32.6)
Lower-manual	225	(27.1)	221	(25.7)	158	(18.2)	266	(29.1)
Non-manual	191	(26.6)	217	(28.3)	163	(20.1)	206	(25.0)
Missing	9	(17.7)	14	(29.1)	10	(20.3)	15	(33.0)
Trusted sources of cancer information								
Healthcare professionals								
Trusting	489	(23.2)	533	(23.9)	420	(18.7)	798	(34.3)
Not trusting	203	(19.5)	242	(22.2)	199	(18.4)	461	(40.0)
Missing	19	(21.2)	10	(14.1)	11	(12.4)	35	(52.4)
Family or friends								
Trusting	277	(20.8)	285	(20.9)	264	(19.3)	551	(39.0)
Not trusting	417	(22.7)	488	(24.7)	357	(18.1)	716	(34.5)
Missing	17	(22.5)	12	(19.0)	9	(12.1)	27	(46.5)
Media								
Trusting	172	(20.5)	197	(22.6)	166	(18.9)	345	(38.0)
Not trusting	505	(22.7)	563	(23.8)	445	(18.9)	861	(34.6)

Missing	34	(18.8)	25	(15.4)	19	(10.8)	88	(54.9)
Internet								
Trusting	339	(23.0)	351	(23.0)	290	(18.6)	574	(35.4)
Not trusting	346	(21.9)	412	(24.4)	317	(18.9)	615	(34.9)
Missing	26	(14.6)	22	(12.0)	23	(13.4)	105	(60.0)
Government/Public Foundations								
Trusting	569	(22.7)	638	(24.1)	488	(18.3)	969	(34.9)
Not trusting	121	(19.9)	132	(20.5)	127	(19.6)	268	(40.0)
Missing	21	(16.9)	15	(13.9)	15	(15.2)	57	(54.0)
Types of tobacco use								
Non-tobacco users	495	(18.3)	665	(23.3)	507	(17.7)	1204	(40.7)
Cigarette users	112	(29.5)	90	(24.0)	91	(23.9)	88	(22.6)
HTP users	104	(62.4)	30	(17.8)	32	(18.8)	2	(1.0)
Types of tobacco use of people living with him/her								
Non-tobacco users	524	(20.5)	611	(22.7)	493	(18.1)	1092	(38.7)
Cigarette users	99	(24.8)	100	(23.4)	77	(18.2)	147	(33.6)
HTP users	86	(34.1)	71	(27.4)	56	(22.4)	42	(16.1)
Missing	2	(10.5)	3	(11.3)	4	(19.5)	13	(58.8)
Cancer history (Their own)								
Yes	50	(14.2)	55	(14.9)	70	(19.4)	179	(51.6)
No	661	(22.8)	730	(24.0)	560	(18.4)	1115	(34.9)
Cancer history (Their family)								
Yes	346	(19.8)	430	(23.5)	346	(18.8)	713	(37.9)
No	295	(24.1)	307	(24.0)	231	(17.8)	463	(34.1)
Not sure	15	(28.1)	10	(17.1)	14	(27.6)	17	(27.3)
Missing	55	(24.7)	38	(15.9)	39	(17.1)	101	(42.3)

HTP, Heated tobacco product.
Participants with missing data for the perception of HTP harmfulness and status of tobacco use were excluded. The proportions shown in parentheses are weighted with the Taylor series linearization method. The percentages may not equal 100% due to rounding to the second decimal place. The percentages shown were the percentage of the perceived HTP harmfulness among respondents who had each explanatory factor. “Tobacco non-users” were those who responded “tobacco product used previously but have not used for more than a month” or “never used” and “Tobacco users” were those who responded “tobacco product use every day” or “use sometimes.”

eTable 3. The characteristics of all the participants by status of tobacco use (n=3,420)

	Non-tobacco users (n=2,871)								Tobacco users (n=549)							
	Less harmful		As/More harmful		Not knowing the harmfulness		Never heard/ Not knowing HTPs well		Less harmful		As/More harmful		Not knowing the harmfulness		Never heard/ Not knowing HTPs well	
	n	%	n	%	n	%	n	%	n	%	n	%	n	%	n	%
Gender																
Male	217	(20.6)	272	(24.1)	221	(19.1)	441	(36.2)	165	(41.3)	91	(22.5)	94	(22.4)	63	(13.8)
Female	278	(16.5)	393	(22.6)	286	(16.6)	763	(44.3)	51	(36.7)	29	(20.0)	29	(21.6)	27	(21.8)
Age groups																
Age 20–39 years	174	(30.4)	171	(28.9)	102	(17.7)	138	(23.0)	53	(49.9)	23	(22.5)	25	(22.5)	6	(5.1)
Age 40–59 years	179	(18.4)	272	(27.2)	201	(20.0)	357	(34.4)	106	(43.7)	58	(24.3)	48	(20.1)	29	(11.9)
Age 60 years or older	142	(11.2)	222	(17.0)	204	(15.9)	709	(56.0)	57	(27.7)	39	(18.3)	50	(25.0)	55	(29.1)
Marital status																
Married	338	(17.6)	458	(23.3)	355	(17.5)	861	(41.6)	160	(43.2)	81	(20.4)	83	(21.7)	60	(14.7)
No spouse	155	(19.6)	206	(23.5)	151	(18.1)	337	(38.8)	56	(34.0)	39	(25.5)	40	(23.3)	30	(17.2)
Missing	2	(19.4)	1	(13.5)	1	(11.8)	6	(55.2)	0	(0)	0	(0)	0	(0)	0	(0)
Education																
Less than college	211	(16.6)	237	(18.6)	227	(17.7)	629	(47.2)	108	(36.0)	67	(22.8)	68	(23.5)	56	(17.8)
College	133	(20.3)	178	(24.9)	115	(16.0)	277	(38.8)	38	(47.2)	20	(24.0)	16	(18.2)	9	(10.6)
University or higher	150	(19.3)	247	(29.0)	164	(19.1)	295	(32.6)	70	(44.7)	33	(19.8)	39	(22.0)	24	(13.4)
Missing	1	(11.4)	3	(41.9)	1	(13.5)	3	(33.2)	0	(0)	0	(0)	0	(0)	1	(100.0)
Household income																
Less than 4 million yen	137	(13.9)	210	(20.8)	168	(16.2)	521	(49.1)	63	(32.9)	43	(22.2)	46	(23.7)	45	(21.1)
4–8 million yen	216	(20.7)	252	(23.3)	200	(18.4)	425	(37.6)	89	(39.4)	51	(21.8)	57	(24.7)	33	(14.1)
More than 8 million yen	128	(21.0)	187	(27.8)	129	(19.9)	215	(31.3)	57	(52.7)	26	(24.7)	17	(15.1)	9	(7.6)
Missing	14	(18.7)	16	(18.9)	10	(11.2)	43	(51.2)	7	(54.9)	0	(0)	3	(19.3)	3	(25.9)
Occupations																
Non-workers	133	(12.8)	205	(18.2)	172	(15.7)	599	(53.3)	35	(28.6)	22	(17.6)	33	(26.5)	34	(27.3)
Manual	67	(20.2)	72	(20.6)	69	(20.5)	150	(38.7)	51	(37.4)	34	(25.8)	25	(19.9)	24	(17.0)
Lower-manual	156	(22.9)	184	(26.5)	129	(18.2)	240	(32.4)	69	(43.9)	37	(22.4)	29	(18.1)	26	(15.6)
Non-manual	133	(22.0)	191	(29.6)	129	(19.1)	201	(29.4)	58	(49.6)	26	(21.8)	34	(25.1)	5	(3.5)
Missing	6	(13.7)	13	(32.1)	8	(17.0)	14	(37.2)	3	(38.2)	1	(13.7)	2	(37.2)	1	(11.0)
Trusted sources of cancer information																
Healthcare professionals																
Trusting	344	(19.3)	460	(24.4)	345	(18.1)	749	(38.2)	145	(43.7)	73	(20.8)	75	(22.0)	49	(13.6)
Not trusting	136	(16.1)	197	(21.9)	152	(17.4)	421	(44.7)	67	(34.3)	45	(23.6)	47	(23.1)	40	(19.1)
Missing	15	(18.8)	8	(11.7)	10	(12.5)	34	(57.1)	4	(42.7)	2	(34.9)	1	(11.2)	1	(11.2)
Family or friends																
Trusting	195	(17.2)	244	(20.8)	219	(18.8)	520	(43.2)	82	(41.3)	41	(21.3)	45	(22.1)	31	(15.4)
Not trusting	287	(19.0)	413	(25.4)	280	(17.1)	657	(38.5)	130	(39.7)	75	(21.7)	77	(22.6)	59	(16.0)
Missing	13	(19.4)	8	(14.3)	8	(12.3)	27	(54.0)	4	(41.4)	4	(47.9)	1	(10.8)	0	(0)
Media																
Trusting	127	(17.2)	175	(23.0)	139	(18.3)	326	(41.5)	45	(41.8)	22	(20.3)	27	(22.2)	19	(15.7)

Not trusting	343	(18.9)	468	(24.1)	352	(18.0)	795	(39.0)	162	(39.8)	95	(22.5)	93	(22.6)	66	(15.0)
Missing	25	(16.0)	22	(14.9)	16	(10.5)	83	(58.6)	9	(41.8)	3	(20.0)	3	(13.4)	5	(24.8)
Internet																
Trusting	236	(18.9)	303	(23.3)	242	(18.2)	544	(39.6)	103	(45.6)	48	(21.6)	48	(20.5)	30	(12.3)
Not trusting	239	(18.4)	344	(24.9)	245	(17.8)	561	(38.9)	107	(37.1)	68	(22.2)	72	(23.9)	54	(16.9)
Missing	20	(13.1)	18	(10.7)	20	(13.2)	99	(63.1)	6	(29.1)	4	(24.1)	3	(15.7)	6	(31.2)
Government/Public Foundations																
Trusting	399	(18.8)	550	(24.5)	405	(17.9)	913	(38.8)	170	(44.2)	88	(21.9)	83	(20.5)	56	(13.4)
Not trusting	79	(16.9)	103	(20.3)	88	(17.2)	236	(45.6)	42	(30.0)	29	(21.1)	39	(27.9)	32	(21.0)
Missing	17	(15.3)	12	(11.8)	14	(15.7)	55	(57.2)	4	(35.4)	3	(37.6)	1	(9.5)	2	(17.5)
Types of tobacco use																
Non-tobacco users	495	(18.3)	665	(23.3)	507	(17.7)	1204	(40.7)								
Cigarette users									112	(29.5)	90	(24.0)	91	(23.9)	88	(22.6)
HTP users									104	(62.4)	30	(17.8)	32	(18.8)	2	(1.0)
Types of tobacco use of people living with him/her																
Non-tobacco users	373	(17.1)	524	(22.8)	403	(17.3)	1031	(42.9)	151	(40.1)	87	(22.5)	90	(22.9)	61	(14.6)
Cigarette users	58	(20.0)	79	(24.8)	54	(17.4)	121	(37.8)	41	(37.5)	21	(19.8)	23	(20.1)	26	(22.6)
HTP users	63	(30.6)	60	(28.5)	46	(22.4)	40	(18.6)	23	(50.2)	11	(22.5)	10	(22.6)	2	(4.7)
Missing	1	(6.7)	2	(8.1)	4	(22.2)	12	(63.1)	1	(36.9)	1	(33.9)	0	(0)	1	(29.2)
Cancer history (Their own)																
Yes	33	(10.6)	50	(15.3)	61	(19.2)	169	(54.9)	17	(41.5)	5	(11.5)	9	(21.2)	10	(25.8)
No	462	(19.2)	615	(24.2)	446	(17.5)	1035	(39.1)	199	(40.2)	115	(22.8)	114	(22.3)	80	(14.7)
Cancer history (Their family)																
Yes	243	(16.3)	371	(23.9)	283	(18.1)	667	(41.7)	103	(39.2)	59	(21.4)	63	(23.0)	46	(16.4)
No	200	(20.0)	256	(24.3)	180	(16.9)	428	(38.8)	95	(42.0)	51	(22.6)	51	(21.4)	35	(13.9)
Not sure	10	(25.2)	7	(14.1)	12	(29.2)	16	(31.5)	5	(40.5)	3	(30.0)	2	(20.4)	1	(9.1)
Missing	42	(22.4)	31	(15.2)	32	(16.1)	93	(46.2)	13	(36.7)	7	(19.3)	7	(22.3)	8	(21.7)

HTP, Heated tobacco product.

Participants with missing data for the perception of HTP harmfulness and status of tobacco use were excluded. The proportions shown in parentheses are weighted with the Taylor series linearization method. The percentages may not equal 100% due to rounding to the second decimal place. The percentages shown were the percentage of the perceived HTP harmfulness among respondents who had each explanatory factor. “Tobacco non-users” were those who responded “tobacco product used previously but have not used for more than a month” or “never used” and “Tobacco users” were those who responded “tobacco product use every day” or “use sometimes.”
